# Improving Patient Access to New Drugs in South Korea: Evaluation of the National Drug Formulary System

**DOI:** 10.3390/ijerph16020288

**Published:** 2019-01-21

**Authors:** Seung-Lai Yoo, Dae-Jung Kim, Seung-Mi Lee, Won-Gu Kang, Sang-Yoon Kim, Jong Hyuk Lee, Dong-Churl Suh

**Affiliations:** 1 College of Pharmacy, Chung-Ang University, Seoul 06974, Korea; firina@naver.com (S.-L.Y.); daejung@daiichisankyo.co.kr (D.-J.K.); hygeia77@hanmail.net (S.-M.L.); dol19@korea.kr (W.-G.K.); jumpingksy@naver.com (S.-Y.K.); 2 Department of Pharmaceutical Engineering, Hoseo University, Asan 31499, Korea

**Keywords:** patient access, reimbursement, risk-sharing agreement, cost-effectiveness analysis, national health insurance

## Abstract

This study reviews and evaluates the national drug formulary system used to improve patient access to new drugs by making reimbursement decisions for new drugs as part of the South Korean national health insurance system. The national health insurance utilizes three methods for improving patient access to costly drugs: risk-sharing agreements, designation of essential drugs, and a waiver of cost-effectiveness analysis. Patients want reimbursement for new drugs to be processed quickly to improve their access to these drugs, whereas payers are careful about listing them given the associated financial burden and the uncertainty in cost-effectiveness. However, pharmaceutical companies are advocating for drug prices above certain thresholds to maintain global pricing strategies, cover the costs of drug development, and fund future investments into research and development. The South Korean government is expected to develop policies that will improve patient access to drugs with unmet needs for broadening health insurance coverage. Simultaneously, the designing of post-listing management methods is warranted for effectively managing the financial resources of the national health insurance system.

## 1. Introduction

Numerous countries have begun prioritizing cost-effectiveness when assessing new medical technologies to more effectively allocate limited health resources given the rising public sector costs associated with an aging population, increased prevalence of patients with chronic diseases, and the introduction of costly new drugs [1,2,3]. It is often difficult to determine reimbursement prices for newly developed drugs when the cost-effectiveness of these drugs in clinical practice remain unclear [4,5]. While pharmaceutical companies are demanding a premium to recover their development costs and fund ongoing investments in research and development (R&D), patients are requesting rapid reimbursement decisions to improve their access to new drugs [6]. Evidence-based evaluation is often difficult for costly new drugs with low demand and no alternatives (this is particularly evident for anticancer drugs and drugs for rare diseases). Therefore, listing new drugs under the public health insurance system without evaluating them can be criticized as a waste of financial resources, whereas designating the drugs as non-reimbursable can lower patient access to them [7,8]. Although recently developed drugs, including cell and gene therapy drugs, have optimal beneficial effects and minimal side effects, their timely delivery can be challenging; there may also be insufficient evidence regarding their efficacy because of the difficulties in recruiting sufficient participants for clinical trials. Moreover, because of the high prices of such drugs, decisions about reimbursement are made while considering a variety of factors, including incentives to improve treatment methods, financial resources available for health insurance, and access to treatment methods [9,10,11,12].

Many countries have begun to employ risk-sharing agreements as a new approach to improve patient access to new drugs. Under these agreements, pharmaceutical companies and insurance payers mutually agree to share the financial burdens or uncertainties regarding clinical outcomes [3,13,14,15]. Countries such as Australia, Great Britain, and Canada have separate organizations or expedited assessment pathways for costly anticancer drugs during the technology assessment and reimbursement decision-making processes [16,17,18,19]. Furthermore, many countries (e.g., Australia, Belgium, Italy, and Great Britain) also possess various drug funds for rare diseases or anticancer drugs to improve patient access to novel drugs [20,21,22,23]. Despite these efforts, many countries have failed to provide possible alternatives for a more comprehensive assessment method that can satisfy all concerned parties.

South Korea implemented a positive listing system policy on 31 December 2006 to allocate financial resources more effectively for medications. Under this new system, only cost-effective drugs can be reimbursed at a premium. The policy also required evidence-based evaluation of new drugs and improved the transparency and consistency in the decision-making process [24,25]. However, such evidence-based evaluations have lowered access to novel drugs which are essential to patients but cannot be subjected to a cost-effectiveness analysis because of challenges in producing clinical evidence and high costs which may not necessarily be reimbursed by insurance [26]. Listing of these drugs to improve patient access would require additional financial resources, thus necessitating the introduction of new policies, such as risk-sharing agreements. This study aims to review the policies currently being implemented for improving patient access to novel drugs by including new drugs under the health insurance system in South Korea and to discuss their outcomes and relevant issues as well as to recommend future policy prospects.

### 1.1. Pharmaceutical Insurance System in South Korea

#### 1.1.1. Drug Expenditures in South Korea

Public health expenditure in South Korea accounts for 7.4% of its gross domestic product (GDP). In general, health expenditure increases as a function of income, which means that Organization for Economic Co-operation and Development (OECD) nations with high GDPs per capita tend to have high health expenditures. When calculated in relation to purchasing power parity, the healthcare expenditure per person in South Korea is $2535, which is lower than the OECD average of $3848 (as of 2015); in fact, South Korea is ranked 25th out of the 35 OECD nations, and therefore South Korean health expenditure is not very high. In contrast, drug expenditures in South Korea for 21.4% of the total healthcare expenditure, which is higher than the OECD average of 16.2%; this puts South Korea as the 6th highest in this category among OECD countries [27]. During South Korea’s decision-making process involving budget allocation, the proportion of drug expenditures constituting the total health budget in comparison with the proportion of drug expenditure of healthcare expenditures in other OECD countries is used as an important index for determining the direction of public health insurance policy. However, use of this proportion is controversial because it can be influenced by healthcare expenditures.

#### 1.1.2. Listing System for the Price of New Drugs in South Korea

**Figure 1:**
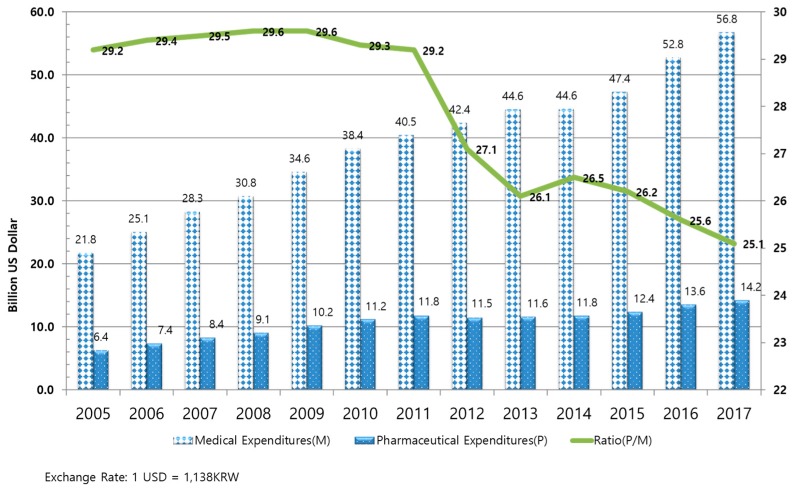
Trends in pharmaceutical expenditures in the South Korean national health insurance. Source: National Health Insurance Service, 2017 Healthcare expenditures, document number:11-B550928-000036-08.

In South Korea, when listing new drugs for coverage under public health insurance, a negative list system had been implemented until the application of a positive listing system on 31 December 2006. When the financial deficit for public health insurance previously peaked in 2001, drug expenditure accounted for 23.5% of total health expenditure. However, this proportion increased to 29.2% in 2005, thereby suggesting issues in drug expenditure management (see Figure 1).

The South Korean government adopted the positive listing system for new drugs in order to allow for more rational management of drug expenditure. Since then, only cost-effective drugs have been listed in the national drug formulary. Moreover, the price–volume agreement system was implemented to manage drug use. Under this system, the prices for drugs whose use increased by 30–60% were reduced by up to 10% according to an agreement between the National Health Insurance Service (NHIS) and the pharmaceutical companies [24,28].

**Figure 2:**
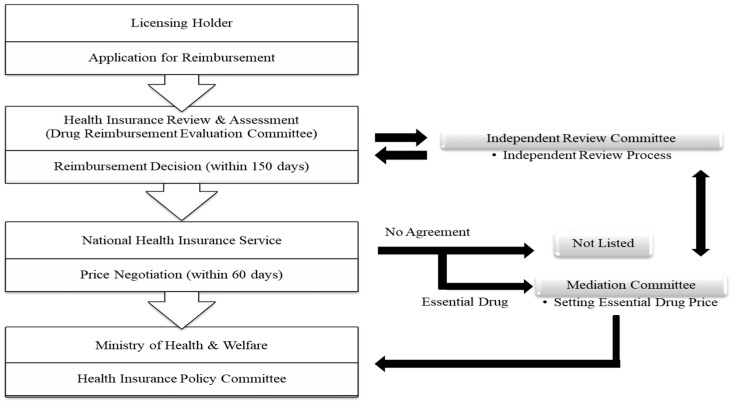
Procedure for listing new drugs in South Korean national health insurance.

The Ministry of Health and Welfare oversees the listing of new drugs as reimbursable drugs. When pharmaceutical companies submit the price of a new drug to the Health Insurance Review and Assessment (HIRA), a Drug Reimbursement Evaluation Committee (DREC) of HIRA evaluates the cost and clinical effectiveness of that drug. For drugs determined to be reimbursable, the NHIS and pharmaceutical companies negotiate drug prices after estimating the financial impact of the addition of a new drug. When the prices are determined, the Health Insurance Policy Deliberative Committee, which serves under the Ministry of Health and Welfare, reviews and approves the prices, after which the drugs are listed as reimbursable drugs [28] (please see Figure 2).

For the evaluation of new drugs, the availability of alternative drugs is initially considered. When alternative drugs are available, the clinical effectiveness of the new drug is evaluated in comparison with these alternative drugs and determined to be non-inferior, superior, or inferior in efficacy. If the new drug is not found to be superior in terms of clinical effectiveness in comparison to the alternative drugs, the drug prices are, in principle, negotiated below the weighted average price of the alternatives for reimbursement. However, the prices of drugs are determined without negotiation if pharmaceutical companies accept 90–95% of the weighted average price of the alternative drugs.

**Figure 3:**
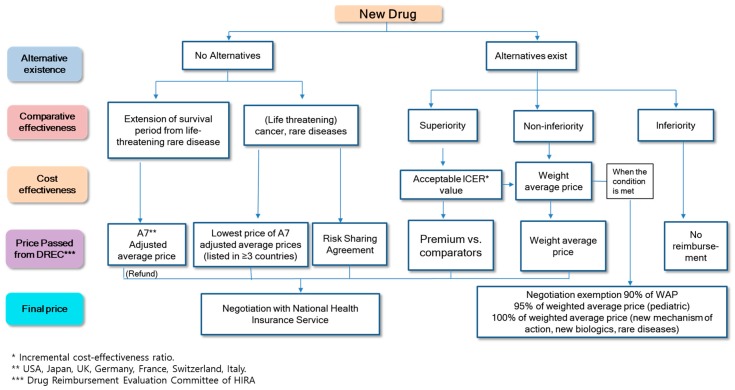
Evaluation procedure for new drug benefits and pricing in Korean national health insurance.

If the drugs are found to be superior to their alternatives, the drug prices are determined based on a cost-effectiveness evaluation. Even when drugs are found to be cost-effective, reimbursement cannot be made unless a financial agreement is reached after negotiation with the NHIS. Drugs considered inferior in comparison with their alternatives remain as non-reimbursable drugs. For new drugs with no available alternatives, drugs that are determined to be essential for treating of patients (thereby classified as essential drugs) by the drug review committee of HIRA are exempted from a cost-effectiveness evaluation. The prices are determined by negotiating with the NHIS based on the prices in A7 countries (i.e., U.S., Japan, Great Britain, France, Germany, Switzerland, and Italy) (see Figure 3).

## 2. Ways of Improving Access to New Drugs in South Korea

In the current South Korean positive listing system, even drugs that are essential for patients may become non-reimbursable if they fail to demonstrate their cost-effectiveness or if the NHIS and pharmaceutical companies fail to reach a financial agreement. It can be difficult for anticancer drugs or orphan drugs to statistically demonstrate their clinical value or cost-effectiveness because of their high costs. Even if drugs are found to be cost-effective, they may remain non-reimbursable if no financial agreement is reached with the insurance payer. In fact, among the drugs evaluated as non-reimbursable between 2007 and 2012, 10 new drugs for advanced-stage or rare diseases became non-reimbursable despite the fact that they were clinically useful and lacked alternatives [29]. Although essential drugs are typically exempted from cost-effectiveness evaluations, the criteria for essential drugs are very specific; consequently, the system was not effective for improving patient access to these new drugs. Accordingly, the South Korean government has introduced new tracks for a risk-sharing agreement as well as a cost-effectiveness analysis waiver to further improve patient access to new drugs.

### 2.1. Designation as Essential Drugs

Drugs are designated by the Drug Review Committee of the HIRA as essential for treatment if it meets all of the following criteria [30]: (a) it has no alternatives (including alternative drugs and treatment methods); (b) it is used for treating serious life-threatening conditions; (c) it is used to treat small patient groups, such as those with rare diseases; and (d) it demonstrates significant improvement in clinical efficacy or survival.

When a drug is designated as essential for treatment, it is exempted from a cost-effectiveness evaluation, and the price for reimbursement is determined by negotiating with the NHIS based on the prices established in the A7 nations. If a pharmaceutical company does not agree to the price of a drug suggested by the NHIS, the drug is not listed as non-reimbursable. In this instance, the reimbursement adjustment committee under the Ministry of Health and Welfare reviews the case, and they may ultimately list the drug as reimbursable. Therefore, listing for reimbursement is considerably easier for essential drugs. Thus far, 10 essential drugs have been listed (see Table 1).

### 2.2. The Risk-Sharing Agreement System

The South Korean government implemented the risk-sharing agreement system in December 2013. Under this system, the government and pharmaceutical companies share the uncertainties regarding the clinical outcomes of new drugs and their influence on the budget, thereby making it easier for drugs to be listed. This risk-sharing agreement system was implemented for adhering to the positive reimbursement of cost-effective drugs while also improving patient access to new drugs and promoting the development of the pharmaceutical industry; it is particularly relevant for cases involving costly anticancer drugs and orphan drugs that lack alternative drugs or treatments.

A drug is eligible for a risk-sharing agreement if it satisfies the following criteria [30]: (a) it should either be an anticancer agent or be used to treat serious, life-threatening conditions and should also lack alternatives or clinically equivalent drugs or treatments; and (b) the drug review committee concludes that further agreement on additional conditions is necessary after considering the severity of the disease, social influences, and other influences on public health. Even when drugs are eligible for a risk-sharing agreement, they are still evaluated according to the same procedures as other drugs. The specifics of the risk-sharing agreement are reflected in the cost-effectiveness evaluation, and the agreement is only reached during the drug price negotiation phase prior to its listing under the public health insurance system.

There are four types of risk-sharing agreements available: refunds, expenditure caps, utilization caps per patient, and refund/expenditure caps. Thus far, 33 drugs have been considered eligible for risk-sharing agreements, and most were finance-based agreements in the form of a refund or expenditure cap, in which an agreement on the refund rate was reached and drug prices corresponding to the refund rate were collected back ex-facto (see Table 2 and Table 3).

### 2.3. Cost-Effectiveness Analysis Waiver System

Although the risk-sharing agreement system was implemented to improve patient access to new drugs, some drugs are not eligible under this system because they are not considered cost-effective. In particular, evidence of cost-effectiveness could not be generated for anticancer agents or orphan drugs [31]. To overcome this shortcoming, the cost-effectiveness analysis waiver system was introduced in February 2015. This system allows drugs that do not satisfy the criteria for essential drugs to be exempted from a cost-effectiveness evaluation if the drug satisfies the strict criteria of the cost-effectiveness analysis waiver system.

For cancer or orphan drugs that are clinically essential but cannot be proven to have a significant improvement in clinical outcomes, only those that are listed in at least three A7 nations can be included (after negotiation with the NHIS) without a cost-effectiveness evaluation. Since September 2016, all new drugs listed under the cost-effectiveness analysis waiver system are required to reach an expenditure cap risk-sharing agreement with the NHIS (i.e., the pharmaceutical companies share the financial risks of the listed drugs). Thus far, 15 new drugs have been listed under the cost-effectiveness analysis waiver system, and 11 of them have an expenditure cap risk-sharing agreement (see Table 4).

## 3. The Impact of Policy on Improvement of Patient Access to New Drugs

Lowering patients’ cost-sharing rates (e.g., 5% for cancer and 10% for rare diseases) can improve patients’ access to new drugs; however, it can also lead to unintended moral hazards [33]. Furthermore, because there is a copayment cap for reimbursable treatment, the maximum copayment per patient cannot exceed an annual limit which is determined based on income (i.e., 1.21–5.09 million Korean won (KRW) which equates to U.S. $1,052–4,426).

Because patients need to pay out-of-pocket for non-reimbursable, it is difficult for patients with cancer or rare diseases to receive treatments with new drugs, which are not listed on the national positive listing formulary because of their high costs. It is imperative to list new drugs on the national drug formulary to reduce patients’ copayment burden and improve their access to these new drugs. However, reduction in the cost-sharing rates inevitably leads to increases in national healthcare budget. It is necessary to continuously monitor the effects of such policies on the financial resources available to ensure stable management of the health insurance system.

### 3.1. The Effects of Policies to Improve Patient Access

The Korean government introduced policies such as listing essential drugs, a risk-sharing agreement, and a cost-effectiveness analysis waiver track to improve patients’ drug access. As of 2017, a total of 15,860 patients have taken advantage of early access to new drugs through these policies, and this number is expected to continuously increase. Total savings by patients amounted to nearly 341 billion KRW; in essence, each patient paid 21 million KRW less as part of copayments compared to what would have been paid without these policies (see Table 5).

### 3.2. The Influence of Policies Aimed at Improving Patient Access to New Drugs on the Financial Resources of Health Insurance

When new drugs are listed as part of the health insurance system, patients’ cost-sharing rates are lowered because majority of the costs are paid by the public health insurance system. In 2017, 62.6 billion KRW were claimed for essential drugs, whereas 260 billion KRW and 39.6 billion KRW were claimed for drugs listed under the risk-sharing agreement system and for cost-effectiveness analysis under the waiver system, respectively. The expenditures are rapidly increasing each year, such that these drugs are thought to exert significant influence on the financial resources available for health insurance (see Table 5). Most of the drugs which have significant government financial burdens are listed under the refund agreement of the risk-sharing agreement system. Therefore, based on the assumption that the refund risk-sharing agreements were made for refunds of 30–50%, at least 78–130 billion KRW would have been collected through paybacks. Conversely, drugs listed under the cost-effectiveness analysis waiver system were often listed under the expenditure cap risk-sharing agreement to control financial risks; thus, their influence is comparatively limited. In 2017, the claimed expenses for drugs listed under the essential drug, risk-sharing agreement, and cost-effectiveness analysis waiver policies accounted for only 0.4%, 1.6%, and 0.2% of the total drug expenses, respectively; therefore, currently, they are not significant threats to the financial resources of the public health insurance system.

## 4. Evaluation of the Policies by Stakeholders

### 4.1. Patients

Patients with serious diseases argue that the time required to list new drugs should be expedited and the listing rate of new drugs should be increased to improve patient access to new drugs [34]. They claim that very few new anticancer drugs are listed under the South Korean health insurance system as compared to the health insurance systems of other advanced countries which makes access to new drugs more difficult. Although many new drugs that were previously non-reimbursable have now become reimbursable under the risk-sharing agreement and cost-effectiveness analysis waiver systems, patients argue that many innovative drugs, including newly developed cell therapy drugs and certain drugs for infectious or rare diseases, should be made eligible under either of these systems to improve drug access.

### 4.2. Insured Persons

Patient advocacy groups agree with the need to increase the reimbursement for advanced cancer and orphan drugs. These groups are comprised of people who pay health insurance costs and monitor whether the government manages health insurance funds appropriately and transparently. They act as vital decision makers in promoting health insurance policies that may need significant financial resources. However, the patient advocacy groups have raised concerns about how listing expensive drugs can induce financial burden and argue that expanding existing systems to improve patient access (i.e., the risk-sharing agreement and cost-effectiveness analysis waiver systems) without thorough preparation for the potential problems in doing so—in particular, problems related to the transparency of drug expenditure and indeterminate clinical efficacy—would only benefit multi-national pharmaceutical companies. The advocate groups have noted that certain expensive anticancer drugs may have questionable clinical efficacy and that patients may expect too much from these drugs because they have not been provided with a detailed explanation regarding the actual drugs’ efficacy or effectiveness.

### 4.3. Pharmaceutical Companies

The pharmaceutical industry argues that South Korea’s complicated procedure for listing drugs and excessively low drug prices restrict competitiveness in the industry. Although it views improved access to new drugs through the various abovementioned policies (e.g., risk-sharing agreement system) as a positive outcome, the pharmaceutical industry still advocates for further improvements in these systems. Many companies claimed that since HIRA requested much lower prices for drugs than the prices established in other countries, they gave up listing the drug in the national drug formulary. Similarly, the excessive government control on drug prices may cause new innovative drugs to be non-reimbursable in South Korea; again, companies argue that this would lower not only patient access to advanced care but also competition in the industry due to low interest in drug development. The pricing policy should be balanced between pricing new medications appropriately in consideration of a national drug budget and allowing for patients’ access to new medication.

Moreover, pharmaceutical companies argue that the scope of application of the essential drug, risk-sharing agreement, and cost-effectiveness analysis waiver systems, which are intended to improve patient access to new drugs, is limited and that the manner in which the systems are currently being implemented does not reflect the intended goal of these systems; therefore, improvements including the expansion of the application scope are necessary [35]. Although the risk-sharing agreement system applies to drugs without alternatives, it still requires a cost-effectiveness evaluation; in addition. Furthermore, because most drugs under the risk-sharing agreements are also a part of the refund agreement, the companies claim that the system should be expanded to include more varied types of agreements.

### 4.4. Insurance Payer (Government)

The government recently reported that patient access to new drugs, particularly to new anticancer drugs, has greatly improved through numerous efforts. According to the government’s analysis conducted between 2014 and 2016 regarding the time required to list new drugs in the national formulary, new drugs without anticancer activity were listed after 269 days of the initial application, while new anticancer drugs were listed after 348 days of the application’s submission. Therefore, more time is needed for listing new anticancer drugs than that required for other new drugs, presumably because anticancer drugs have more issues related to risk-sharing agreements and cost effectiveness studies as opposed to other classes of new drugs. The South Korean government has sought to decrease the listing time of new anticancer drugs to 240 days. Moreover, the listing rate of new anticancer drugs (i.e., the percentage of drugs that were listed among all drugs applied for listing) increased from 43% between 2008 and 2013 to 53% between 2014 and 2016, and the number of listed drugs increased from 3.5 drugs per year between 2008 and 2013 to 11 per year between 2014 and 2015. Thus, the government has deemed the policies directed towards improving access to new drugs as effective. The government is further striving to improve the risk-sharing agreement and cost-effectiveness analysis waiver systems to further optimize the listing rates.

The argument put forward by the pharmaceutical industry—that the prices for new drugs are lower in South Korea than in other countries—has been countered by the South Korean government; the government states that there are various discount or refund agreements hidden behind the listed prices in other countries. Furthermore, because the South Korean government views the listed prices in other countries as an inaccurate reflection of the actual drug purchase price after adjustments (i.e., post-rebate), they determine drug prices according to the prices of available alternatives and the results of cost-effectiveness analyses rather than the prices adopted in other countries. Patient access to new drugs and industrial development can suffer if innovative new drugs essential for patient treatment are not listed in the national drug formulary or if there is a delay in listing such drugs, thus, the government is willing to develop more flexible regulations for the listing of new drugs as well as implement a more thorough post-listing management strategy [36,37].

## 5. Discussion

In 2017, the South Korean government announced a proposal to expand the coverage of medical services and drugs, which involved providing reimbursement for all currently non-reimbursable drugs. Under this system, reimbursement is provided if a certain level of cost-effectiveness is achieved, but different copayment rates are applicable depending on the disease characteristics and patients’ needs. The system aims to relieve financial burdens by increasing patients’ copayment rates for drugs whose cost-effectiveness has not been verified while also increasing patient access to new drugs. For example, instead of the current rate of 5% copayment for cancer drugs, a higher copayment can be implemented to relieve the government’s financial burden resulting from the rapid reimbursement process to improve patients’ access to new medications [38].

The government is currently discussing a “pre-listing and post-cost effectiveness evaluating system,” according to which drugs will be listed under the health insurance system at temporary prices without undergoing a cost-effectiveness evaluation, and reimbursement and prices will be determined later through a follow-up evaluation process. This method would be applied to drugs that have high demand but also pose an economic burden, and the costs spent until evaluation would, in part, be reclaimed for drugs which were ultimately not found to be cost-effective. However, it is still uncertain whether this system will be implemented.

Whether the clinical efficacy evaluated at the time of listing new drugs remains consistent with their actual clinical effectiveness contributes to the uncertainty in the decision making process about insurance coverage and drug prices. Therefore, it is necessary to verify and re-evaluate whether clinical outcomes remain consistent at the initially appraised level of impact. In particular, the societal need to re-evaluate the clinical utility of drugs which are in high demand for patient care and that represent a substantial financial burden, such as recently listed targeted anticancer drugs and cancer immunotherapy drugs, has increased [39]. Because the analysis of health insurance claims data offers limited information for the evaluation of actual clinical efficacy, methods of preparing a system to analyze the cost-effectiveness by investigating the clinical efficacy and cost in real-world clinical settings are being sought.

### 5.1. Policy Suggestions

#### 5.1.1. Increase the Transparency and Rationality of Cost-Effectiveness Analysis

Although performing a review of the data obtained from cost-effectiveness analyses conducted in South Korea may be a time-consuming process, it is nevertheless considered the most important factor for decision-making. Improvements to reduce the time costs of reviewing such studies and solve issues regarding the transparency and efficacy of the review process are warranted.

The acceptable threshold for the incremental cost-effectiveness ratio (ICER) for most new drugs is often below 25,000,000 KRW (USD 21,968, which is equivalent to Korea’s per capita GDP) per quality-adjusted life year. This threshold value was created based on per capita GDP in Korea at the time in 2016. Since then, the value has not been adjusted for changes in per capita GDP in Korea. Thus, adjustments should be made to the acceptable threshold value to account for changes in per capita GPD, and more flexible ICER thresholds for reimbursement should be implemented based on diseases’ severities or societal impacts to improve patient access to care. Currently, for a limited number of anticancer drugs and orphan drugs, a flexible incremental cost-effectiveness ratio threshold of up to 50,000,000 KRW (USD 43,936; two times GDP) per quality-adjusted life year has recently begun to be applied.

The WHO recommended a threshold value of 1–3 times a country’s per capita GDP but they did not provide additional justification for this value [40]. The decision process of medication selection based on ICERs often raises questions about a society’s willingness to pay for increased QALYs. For example, National Institute for Health and Care Excellence (NICE) in the UK has explicitly used a willingness to pay threshold ranging from £20,000/QALY (U.S. $25,000) to £50,000 (U.S. $62,500) for life-threatening conditions. Australia does not have an explicit threshold but accepted new medications which have thresholds ranging from AUD45,000 to AUD60,000 per QALY (U.S.$31,500–U.S.$42,500) [41]. The U.S. uses a wide range of thresholds from U.S. $50,000/QALY to $150,000/QALY depending on individual preferences or disease severity.

#### 5.1.2. Improvement of the Risk-Sharing Agreement System

For chronic diseases such as osteoarthritis, and acute infectious diseases such as hepatitis C, only symptom relief or temporary treatments were available in the past. However, recent improvements have led to the development of innovative new drugs that can increase survival and possibly lead to a complete recovery; hence, the frequency of requests pertaining to the listing of these drugs has increased. Compared to other countries, South Korea has a more limited scope for entering into risk-sharing agreements because only certain anticancer or orphan drugs are listed under this system. Most drugs under the risk-sharing agreement system are also associated with refund agreements because of considerable uncertainty that results from the paucity of explicit guidelines on outcome-based risk-sharing agreements.

There is a lack of agreement among the government, manufacturers, and patient advocate groups on the eligibility criteria of a risk-sharing agreement. Patient advocate groups are concerned about the lack of transparency of the list price and the actual price after rebate. The risk-sharing agreement application should be expanded to include medications that do not have alternatives or diseases such as acute attacks of hereditary angioedema, moderate to severe asthma, systemic lupus erythematosus, and cryopyrin-associated periodic syndromes. In addition, the Korean government should provide clearer guidelines on how various forms of outcome-based risk-sharing agreements can be employed for each drug.

#### 5.1.3. Improvement of the Drug Price Listing Process

The prices of new drugs in South Korea are determined based on two steps: first, the cost-effectiveness evaluation (i.e., suitability for reimbursement) is performed by HIRA followed by a financial agreement (i.e., negotiation on drug prices based on budget impact analysis and referencing price, and price-volume agreement, etc.) with the NHIS. There is mounting criticism that these two-step procedures lowered the viability and transparency of the drug pricing system [42,43]. As a result, some experts suggested that the procedures followed by HIRA and NHIS for determining drug prices should be combined. However, a rebuttal for this argument is that the current two-step approach is useful for checking and balancing the powers of HIRA and NHIS in the process of listing new medications; as such, it is unlikely that the tasks will ever be combined. Therefore, to ensure that each organization achieves its own goals, the roles of the HIRA and NHIS should be clearly defined.

To improve patient access to care as well as determine the appropriate value of novel medications, a package negotiation system with the NHIS—where prices are negotiated for a group of drugs with similar purposes made by one company—could be implemented rather than negotiating individual prices for each drug. This package system could improve the current system and prevent drugs from failing to be listed because of the lack of a financial agreement, despite the drugs being considered suitable for reimbursement. Varying the negotiation methods to more effectively manage financial resources may also be a way for insurance providers to better fulfill their roles [44]. The Korean government recently implemented a new policy to expedite the review process period of NHIS from 60 days to 30 days. If the efficacy of a new medication is similar to those of comparators’ and a manufacturer accepts a price lower than the weighted average price in the market, the negotiation process is skipped entirely.

#### 5.1.4. The Necessity of External Financial Resources for Orphan and Anticancer Drugs Independent of the NHIS

Countries such as the UK and Australia have separate cancer drug funds to provide patients with access to drugs that have clinically plausible potential with additional data but have not yet been appraised [20,21,22,23]. The funds improve patients’ access to new innovative drugs to treat rare diseases or cancers that satisfy specific criteria while adhering to the rules of the health insurance system. Because South Korea does not have a similar policy and relies only on health insurance premiums, it is exceedingly difficult to provide reimbursement for costly cancer or orphan drugs that have a high societal demand and need but uncertain clinical outcomes. Covering these drugs under the health insurance system would contradict the principles of universal insurance and may lower equity. Therefore, additional funding sources outside of health insurance should be procured.

## 6. Conclusions

The national health insurance system in South Korea has gradually improved quality medical services and access to medications at reasonable prices. However, the health insurance coverage rate is currently only 63%, which falls well short of the OECD average of 80%. In order to improve the coverage rate, the government recently announced a roadmap to provide reimbursement for all currently non-reimbursable treatments and medications. It is expected to make significant changes in the scope, reimbursement type, and price determination processes.

Several policies have been implemented after the introduction of the positive formulary listing system in 2007, and both cost-effectiveness evaluations by HIRA and financial agreements with the NHIS have played, and will continue to play, vital roles with regards to listing of drugs in the national formulary. Continued improvement in current policies and the introduction of new policies is likely to improve patient access to new and advanced drugs in South Korea, and recommendations to implement financial management policies to ensure sustainability of financial resources for insurance will further balance these two aspects.

## Figures and Tables

**Table 1:** Drugs evaluated by HIRA in South Korea.

Brand Name	Active Substance	Indication	Evaluation Year
Cystadane	Betaine anhydrous	Homocystinuria	2007
Sprycel	Dasatinib	Leukemia	2007
Elaprase	Idursulfase	Mucopolysaccharidosis type II	2008
Naglazyme	Galsulfase	Mucopolysaccharidosis type VI	2008
Myozyme	Alglucosidase alpha	Pompe disease	2008
Zavesca	Miglustat	Gaucher’s disease	2009
Inovelon	Rufinamide	Lennox–Gastaut syndrome	2010
Remodulin	Treprostinil	Pulmonary hypertension	2010
Soliris	Eculizumab	Paroxysmal nocturnal hemoglobinuria	2011
Carbaglu	Carglumic acid	Hyperammonemia	2014

**Table 2:** Drugs listed with a risk-sharing agreement in South Korea (2014.1.–2018.6.).

Product (Active Substance)	Indication	Risk-Sharing Agreement Type	Cost Effectiveness Analysis (CEA)
Eboltra (clofarabine)	Acute lymphoblastic leukemia	Coverage with evidence development	X
Erbitux (cetuximab)	Colorectal cancer	Refund	O
Revlimid (lenalidomide)	Multiple myeloma	Refund	X
Xtandi (enzalutamide)	Prostate cancer	Refund	O
Xalkori (crizotinib)	Non-small cell lung carcinoma	Refund	O
Pirespa (pirfenidone)	Idiopathic pulmonary fibrosis	Refund	O
Soliris (eculizumab)	Paroxysmal nocturnal hemoglobinuria	Refund	X (essential drug)
Caprelsa (vandetinib)	Thyroid gland cancer	Expenditure cap	X (waiver of CEA)
Naglazyme (galsulfase)	Mucopolysaccharidosis	Refund	X (essential drug)
Stivarga (regorafenib)	Gastrointestinal tumors	Refund	O
Vimizim (elosulfase alfa)	Morquio syndrome	Expenditure cap	X (waiver of CEA)
Diterin (sapropterin)	Phenylketonuria	Expenditure cap	X (waiver of CEA)
Pomalyst (pomalidomide)	Multiple myeloma	Refund	O
Defitelio (defibrotide)	Hepatic veno-occlusive disease	Expenditure cap	X (waiver of CEA)
Perjeta (pertuzumab)	Breast cancer	Utilization cap per patient	O
Zelboraf (vemurafenib)	Melanoma	Expenditure cap	X (waiver of CEA)
Kadcyla (trastuzumab emtansine)	Breast cancer	Utilization cap per patient	O
Keytruda (pembrolizumab)	Non-small cell lung carcinoma	Refund/ Expenditure cap	O
Opdivo (nivolumab)	Non-small cell lung carcinoma	Refund/ Expenditure cap	O
Lynparza (olaparib)	Ovarian cancer	Expenditure cap	X (waiver of CEA)
Meqsel (trametinib)	Melanoma	Expenditure cap	X (waiver of CEA)
Ibrance (palbociclib)	Breast cancer	Refund	O
Olita (olmutinib)	Non-small cell lung carcinoma	Expenditure cap	X (waiver of CEA)
Tagrisso (osimertinib)	Non-small cell lung carcinoma	Refund	O
Rafinlar (dabrafenib)	Melanoma	Expenditure cap	X (waiver of CEA)
Alecensa (alectinib hydrochloride)	Non-small cell lung carcinoma	Expenditure cap	X (waiver of CEA)
Tecentriq (atezolizumab)	Non-small cell lung carcinoma	Expenditure cap	X (waiver of CEA)
Sylvant (siltuximab)	Castleman’s disease	Expenditure cap	X (waiver of CEA)
Kyprolis (carfilzomib)	Multiple myeloma	Refund	O
Lartruvo (olaratumab)	Soft tissue tumors and sarcomas	Expenditure cap	X (waiver of CEA)
Iclusig (ponatinib)	Leukemia	Expenditure cap	X (waiver of CEA)
Imbruvica (ibrutinib)	Mantle cell lymphoma	Expenditure cap	X (waiver of CEA)
Cyramza (ramucirumab)	Gastric cancer	Refund	O

(Note) Of the 33 total medications, two (Pirespa and Revlimid) have been terminated due to generic drug registration.

**Table 3:** Types of risk-sharing agreements and drug categorization in South Korea.

Risk-Sharing Agreement Type	Cancer Drug	Cancer/Orphan Drug	Orphan Drug	Total Number of Drugs	(%)
Coverage with additional evidence	0	1	0	1	(3.0)
Expenditure cap	2	10	3	15	(45.5)
Refund	6	3	3	12	(36.4)
Utilization cap per patient	2	1	0	3	(9.1)
Refund/Expenditure cap	2	-	-	2	(6.1)
Total	12	15	6	33	(100.0)

**Table 4:** Drugs with waiver policy of cost-effectiveness analysis requirement in South Korea.

Product	Active Ingredient	Indication	Reimbursed Year	Risk-Sharing Agreement Type
Caprelsa	Vandetanib	Thyroid gland cancer	2015	Expenditure cap
Adcetris	Brentuximab vedotin	Hodgkin’s lymphoma	2016	Not applied
Imbruvica	Ibrutinib	Mantle cell lymphoma	2016	Not applied
Vimizim	Elosulfase alfa	Morquio syndrome	2016	Expenditure cap
Zykadia	Ceritinib	Non-small cell lung carcinoma	2016	Not applied
Blincyto	Blinatumomab	Lymphocytic leukemia	2016	Not applied
Diterin	Sapropterin	Phenylketonuria	2017	Expenditure cap
Defitelio	Defibrotide	Hepatic veno-occlusive disease	2017	Expenditure cap
Zelboraf	Vemurafenib	Melanoma	2017	Expenditure cap
Lynparza	Olaparib	Ovarian cancer	2017	Expenditure cap
Meqsel	Trametinib	Melanoma	2017	Expenditure cap
Olita	Olmutinib	Non-small cell lung carcinoma	2017	Expenditure cap
Sylvant	Siltuximab	Castleman’s disease	2018	Expenditure cap
Lartruvo	Olaratumab	Soft tissue tumors and sarcomas	2018	Expenditure cap
Iclusig	Ponatinib	Leukemia	2018	Expenditure cap

Source: HIRA, list of reimbursable drugs [32].

**Table 5:** Impact of policy schemes on patient access improvement.

Category		2015		2016		2017	
		Amount	(%)	Amount	(%)	Amount	(%)
Total drug expenditures	(million KRW)	14,098,500	(100)	15,428,600	(100)	16,209,800	(100)
	(million USD)	12,389		13,558		14,244	
Essential drugs	(million KRW)	53,522	(0.38)	60,753	(0.39)	62,604	(0.39)
	(million USD)	47.0		53.4		55.0	
	No. of patients	1506		1719		1812	
Risk-sharing agreement drugs	(million KRW)	103,518	(0.74)	161,358	(1.05)	260,360	(1.61)
	(million USD)	91.0		141.8		228.8	
	No. of patients	5125		7861		13,112	
CEA waiver drugs	(million KRW)	58	(0.00)	13,516	(0.09)	39,672	(0.25)
	(million USD)	0.05		11.9		34.9	
	No. of patients	12		343		936	
Total amount saved by patients *	Total (million KRW)	148,033	(1.05)	221,414	(1.44)	341,140	(2.10)
	Total (million USD)	130.1		194.6		299.8	
	Amount per person (million KRW)	22.3		22.3		21.5	
	Amount per person (million USD)	0.02		0.02		0.02	

Source: National Health Insurance Service, 2018 Health Insurance Claims Data; Abbreviation: CEA: cost effectiveness analysis, KRW: Korean won; USD: U.S. dollar; No.: number; Exchange Rate: 1 USD = 1138 KRW; * The “total amount saved by patients” does not equal the sum of the previous categories because some medications are included in more than one category.
